# Neighborhood greenness in midlife associated with cognitive decline in later life: The Multi‐Ethnic Study of Atherosclerosis

**DOI:** 10.1002/alz70860_099334

**Published:** 2025-12-23

**Authors:** Lilah M Besser, Jana A. Hirsch, Timothy M. Hughes, Peter James, Marcia Pescador Jimenez, Kari Moore, Joel D. Kaufman, Ana V. Diez‐Roux

**Affiliations:** ^1^ University of Miami Miller School of Medicine, Boca Raton, FL, USA; ^2^ Drexel University, Philadelphia, PA, USA; ^3^ Wake Forest University School of Medicine, Winston‐Salem, NC, USA; ^4^ University of California Davis Center for Occupational and Environmental Health, Davis, CA, USA; ^5^ Boston University School of Public Health, Boston, MA, USA; ^6^ University of Washington, Seattle, WA, USA

## Abstract

**Background:**

Green neighborhoods (e.g., with parks, gardens, street trees) offer spaces for physical activity, social engagement, and stress reduction, factors associated with reduced Alzheimer's disease and related dementia (ADRD) risk. Greener neighborhoods may be particularly impactful on these health behaviors during midlife when ADRD pathology develops. Yet, few studies have investigated associations between midlife greenness exposure and late‐life cognition. In this study, we investigated associations between neighborhood greenness in midlife and cognitive decline in later life.

**Methods:**

We used data on 2,881 participants in the population‐based Multi‐Ethnic Study of Atherosclerosis (MESA), restricting to those with address history covering midlife (45‐54 years old). Geocoded addresses (1980‐2009) were used to derive measures of midlife neighborhood greenness in the 1,230m buffer surrounding the residence using the normalized difference vegetation index (NDVI) based on LANDSAT satellite imagery (range: 0‐1, closer to 1=greener). We calculated the 10‐year mean neighborhood NDVI value during the summertime (∼July 1) for the midlife period. Global cognition (Cognitive Abilities Screening Instrument, CASI) and processing speed (Digit Symbol (DSC)) were measured three times spanning ∼10 years. Participants were ≥55 years old at their baseline cognitive test (Exam 5). Multivariable linear mixed effects regression tested associations between midlife 10‐year mean NDVI and longitudinal change in CASI and DSC z‐scores (based on sample mean and SD at baseline cognitive visit). Models controlled for site and time from Exam 5, as well demographics (e.g., age, ethnoracial group, gender, education), neighborhood socioeconomic status, and comorbidities (e.g., hypertension, diabetes), all measured at Exam 5. Greenness×time interaction terms tested whether midlife greenness is associated with cognitive decline.

**Results:**

Participants were 65.8±6.0 years old; 53% were women; and 28% were Black, 10% Chinese, 21% Hispanic, and 42% non‐Hispanic White. Individuals in the greenest neighborhoods in midlife (versus least green) (i.e., NDVI=1 versus 0) had slower annual decline in CASI (estimate=0.038, 95% CI=0.009, 0.066) and DSC z‐scores (estimate=0.050, 95% CI=0.021, 0.080) in later life.

**Conclusion:**

In a geographically and ethnoracially diverse US cohort, we found that living in greener neighborhoods in midlife was associated with slower decline in global cognition and processing speed over time in later life.

